# The role of peak serum estradiol level in the prevention of multiple pregnancies in gonadotropin stimulated intrauterine insemination cycles

**DOI:** 10.1038/s41598-022-23470-9

**Published:** 2022-11-15

**Authors:** Pierre-Emmanuel Bouet, Mariette Bruand, Kevin Bellaïche, Bruno Vielle, Guillaume Legendre, Philippe Descamps, Romain Corroenne, Pascale May-Panloup, Hady El Hachem

**Affiliations:** 1 Department of Reproductive Medicine, Angers University Hospital, 4 Rue Larrey, 49000 Angers, France; 2 Clinical Research Center, Angers University Hospital, Angers, France; 3 Department of Obstetrics and Gynecology, Lebanese American University, Beirut, Lebanon

**Keywords:** Infertility, Predictive markers

## Abstract

The objective was to assess whether the measurement of serum estradiol (E_2_) level on trigger day in controlled ovarian stimulation with intrauterine insemination (COS-IUI) cycles helps lower the multiple pregnancy (MP) rate. We performed a unicentric observational study. We included all patients who underwent COS-IUI and had a subsequent clinical pregnancy (CP) between 2011 and 2019. Our main outcome measure was the area under Receiver-Operating Characteristic (ROC) curve. We included 455 clinical pregnancies (CP) obtained from 3387 COS-IUI cycles: 418 singletons, 35 twins, and 2 triplets. The CP, MP, and live birth rates were respectively 13.4%, 8.1% and 10.8%. The area under ROC curve for peak serum E_2_ was 0.60 (0.52–0.69). The mean E_2_ level was comparable between singletons and MP (260.1 ± 156.1 pg/mL vs. 293.0 ± 133.4 pg/mL, *p* = 0.21, respectively). Univariate and multivariate logistic regression analysis showed that E_2_ level was not predictive of MP rate (aOR: 1.13 (0.93–1.37) and 1.06 (0.85–1.32), respectively). Our study shows that, when strict cancelation criteria based on the woman’s age and follicular response on ultrasound are applied, the measurement of peak serum E_2_ levels does not help reduce the risk of MP in COS-IUI cycles.

## Introduction

In the past 40 years, and since the advent of assisted reproductive technologies (ART), the number of multiple pregnancies (MP) has doubled in developed countries, going from 8–10‰ to 16–20‰^1^. This is mainly the consequence of controlled ovarian stimulation (COS) with oral medications and injectable gonadotropins leading to multiple follicular growth and ovulation, and the transfer of multiple embryos in in vitro fertilization (IVF) cycles^2,3^. The significant increase in the number of MP is considered a major public health problem, mainly because of the increased risk of prematurity and the subsequent short, middle and long-term complications^4^. In the past 20 years, several countries have implemented a single-embryo transfer (SET) policy in order to decrease the number of MP following IVF^5^, leading in many cases to a 10% decrease in the MP rate, going from 26% in 1999 to 15% in 2015^6,7^. However, over that same period, the rate of MP following COS, with or without intrauterine insemination (IUI), has remained stable at around 10–15%^6,7^.

Several risk factors have been associated with the risk of MP in COS cycles, with or without IUI: the woman’s age, the number of preovulatory follicles > 10 or 14 mm on ultrasound (US), and the serum estradiol (E_2_) level on the day of ovulation triggering^8–12^. In the past decade, many algorithms taking into account these factors have been put in place in order to decrease the risk of MP, but without any significant success^12–14^. To date, there is no consensus on the best method for monitoring COS cycles followed by IUI (COS-IUI). The most commonly used is serial US monitoring of follicular growth, with the use of strict cancelation criteria based on the woman’s age and number of preovulatory follicles^15^. Some centers add serum E_2_ measurement to US monitoring to decrease the risk of MP, with the arguments being: (1) a very high E_2_ level (> 862 pg/mL, > 1000 pg/mL or > 2000 pg/mL, depending on studies) is a good predictive factor of MP^10,12,16^; (2) serum E_2_ levels can help in the decision making for ovulation trigger in equivocal cases, and in adjusting any error in US measurement of follicular growth; (3) measurement of serum E_2_, progesterone and Luteinizing Hormone (LH) levels allows the diagnosis of any premature ovulation in stimulated cycles, thus advancing the timing of IUI.

On the other hand, US monitoring alone can be sufficient since: (1) serum E_2_ level is directly correlated to the number of growing follicles, with intrafollicular and serum E_2_ levels steadily increasing with the follicular growth^17,18^; (2) it is extremely rare to have a discordance between follicle number and size and the serum E_2_ level^12^; (3) the diagnosis of any premature ovulation and the subsequent advancement of the IUI timing has not been shown to improve cycle outcomes, especially if the couple is sexually active during the cycle^19^.

Based on these arguments, we aimed to assess whether the systematic measurement of serum E_2_ levels on trigger day allows to decrease the MP rate in COS-IUI cycles when strict cancelation criteria are used.

## Results

**Figure 1:**
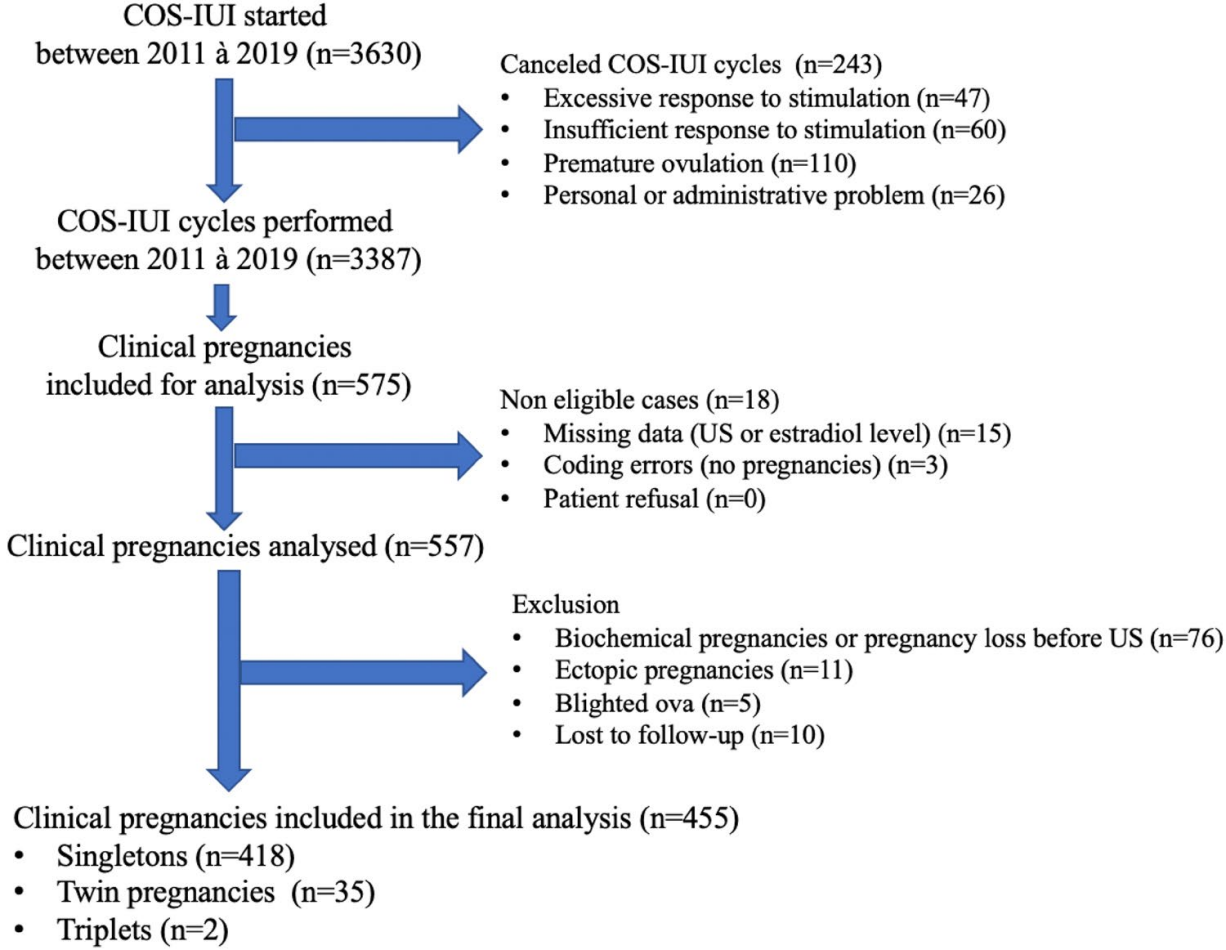
Study flow chart.

Between 2011 and 2019, 3630 COS-IUI cycles were started at our center. The flow chart in Fig. 1 shows the distribution of the population.

### Population characteristics

The population characteristics are shown in Table 1. The mean age of women was 31.5 ± 4.8 years, and in 61% of cases, infertility was unexplained. The mean duration of stimulation was 8.2 ± 4.9 days, and the mean total FSH dose used per cycle was 744.4 ± 689.8 IU. On trigger day, mean serum E_2_ level was 263.3 ± 154.5 pg/mL and the mean number of follicles ≥ 10 mm and ≥ 14 mm was 2.3 ± 1.2 and 1.6 ± 0.8, respectively.

**Table 1:** Baseline characteristics of patients/cycles who achieved a clinical pregnancy.

	Clinical pregnancies (N = 455)
Age (years)	31.5 ± 4.8
Body Mass Index (kg/m^2^)	24.7 ± 5.7
Primary infertility	296 (69)
Duration of infertility (years)	2.9 ± 1.9
Causes of infertility*	
Dysovulatory	79 (17.4)
Endometriosis	15 (3.3)
Moderate male factor	25 (5.5)
Low ovarian reserve	16 (3.5)
Unexplained	310 (68.1)
Serum estradiol E_2_ (pg/ml)	43.8 ± 33.3
Baseline FSH (IU/L)	7.0 ± 4.1
Baseline LH (IU/L)	5.6 ± 3.6
Duration of stimulation (days)	8.2 ± 4.9
Total dose of FSH per cycle (IU)	744.4 ± 689.8
E_2_ level on trigger day (pg/ml)	263.3 ± 154.5
Progesterone level on trigger day (ng/ml)	0.41 ± 0.6
LH level on trigger day (IU/L)	7.45 ± 9.9
Number of follicles ≥ 10 mm on trigger day	2.3 ± 1.2
Number of follicles ≥ 14 mm on trigger day	1.6 ± 0.8
Number of motile sperm inseminated (millions)	10.3 ± 7.1

Data are expressed as n (%) percentage or mean + / − standard deviation.

*Some couples could have had multiple causes of infertility.

### Outcomes

The outcomes of the COS-IUI cycles are shown in Table 2.

**Table 2:** Outcomes of COS-IUI cycles performed during the study period.

Clinical pregnancy rate	13.4% (455/3387)
Miscarriage rate	14.5% (66/455)
Twin pregnancy rate	7.7% (35/455)
Higher order pregnancy rate*	0.4% (2/455)
Live birth rate	10.8% (367/3387)

Data are expressed as percentage (n/total).

*Defined as the presence of more than two embryos on the ultrasound at 7 weeks GA.

The two triplets occurred in women aged 26 and 28 years treated for unexplained infertility, who received in 6 and 7 days of stimulation a total dose of 450 and 525 IU, respectively. On trigger day, they had 2 and 3 follicles ≥ 10 mm, and a serum E_2_ of 257 and 355 pg/mL, respectively.

**Figure 2:**
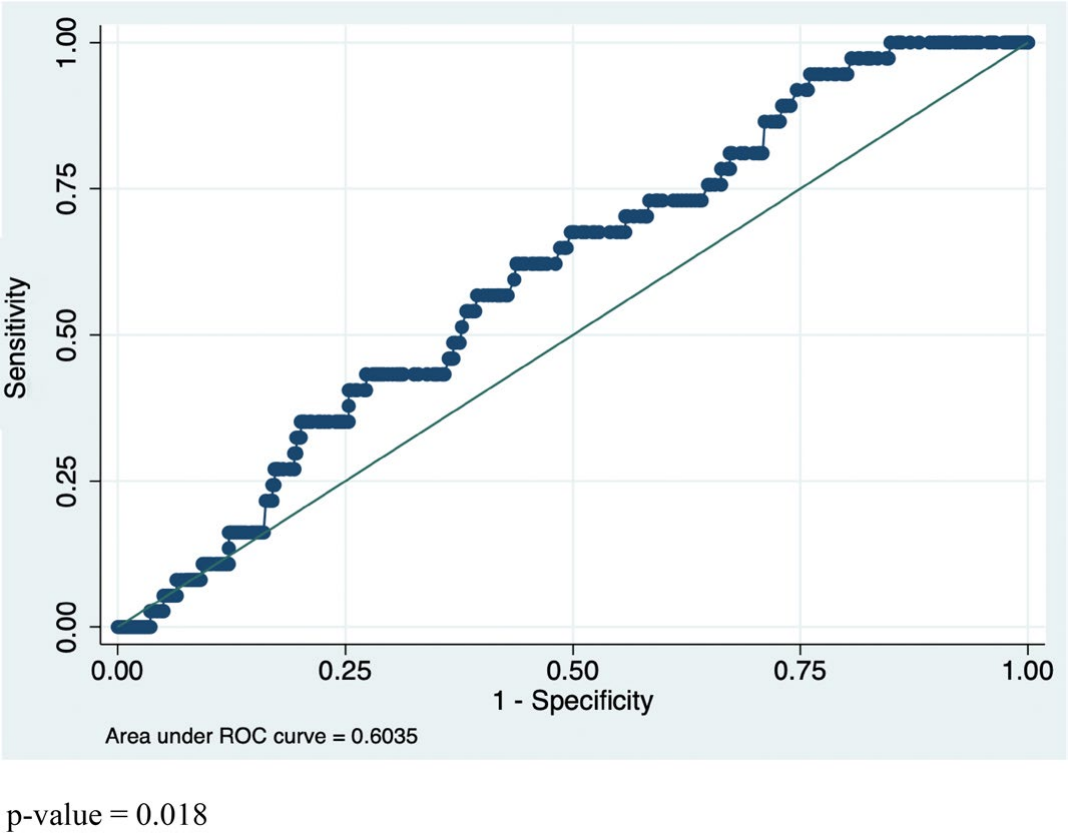
ROC curve for peak serum estradiol.

The area under ROC curve for peak serum estradiol was 0.60 (0.52–0.69) (Fig. 2). Serum E_2_ level on trigger day was < 500 pg/mL in 89% (33/37) of cases of MP. There were no cycles performed with a peak serum E_2_ level ≥ 1000 IU pg/mL.

We performed a univariate analysis to assess the factors predictive of MP. We found that the number of follicles ≥ 14 mm on trigger day was significantly higher in the MP group compared to the singleton group (1.8 ± 0.8 vs 1.5 ± 0.8, *p* = 0.04, respectively) (Table 3). However, peak serum E_2_ level was not predictive of MP (OR = 1.13 (0.93–1.37)). Multivariate logistic regression showed an adjusted OR of 1.06 (0.85–1.32) for peak E_2_ level, 1.39 (0.94–2.05) for the number of follicles ≥ 14 mm (Table 4), and 1.17 (0.87–1.58) for the number of follicles ≥ 10 mm (supplementary data).

**Table 3:** Univariate analysis to assess the factors predictive of MP (GEE model).

	Singleton pregnancy (N = 418)	Multiple pregnancy (N = 37)	*p*-value	OR (IC95%)
Age (years)	31.5 ± 4.8	31.4 ± 4.3	0.89	0.99 (0.93–1.07)
Total treatment dose (IU)	730.6 ± 677	900.0 ± 809	0.16	1.03^c^ (0.99–1.07)
Duration of treatment (days)	8.1 ± 4.7	9.4 ± 6.9	0.13	1.04 (0.99–1.10)
**Causes of infertility**^a^				
Dysovulatory	70 (16.7)	9 (24.3)	0.25	1.60 (0.72–3.53)
Endometriosis	14 (3.3)	1 (2.7)	0.83	0.80 (0.10–6.27)
Moderate male factor	22 (5.3)	3 (8.1)	0.47	1.59 (0.45–5.58)
Low ovarian reserve	15 (3.6)	1 (2.7)	0.78	0.75 (0.09–5.81)
Unexplained	288 (68.9)	22 (59.5)	0.24	0.66 (0.33–1.32)
Mean E2 (pg/mL)	260.1 ± 156.1	293.9 ± 133.4	0.21	1.13^c^ (0.93–1.37)
Number of follicles ≥ 10 mm on trigger day	2.2 ± 1.2	2.6 ± 1.2	0.08	1.24 (0.97–1.59)
Number of follicles ≥ 14 mm on trigger day	1.5 ± 0.8	1.8 ± 0.8	0.04	1.44 (1.02–2.02)
Male factor^b^	10.4 ± 7.2	9.7 ± 4.8	0.63	0.98 (0.93–1.05)

^a^Some couples could have had multiple causes of infertility.

^b^Concentration of sperm with progressive motility (all > 3 × 10^6^ /mL according to inclusion criteria.

^c^Odds ratio was calculated for a variation of 100 units of the corresponding variable.

**Table 4:** Multivariate logistic regression for MP (twin and higher order multiple pregnancies) (multivariate GEE model).

	aOR (95% CI)	*p*-value
Duration	1.05 (0.97–1.14)	0.23
Doses	0.99 (0.94–1.06)	0.90
Estradiol level	1.06 (0.85–1.32)	0.60
Follicles ≥ 14 mm on trigger day	1.39 (0.94–2.05)	0.10

We found a moderate correlation between the number of follicles ≥ 10 mm and ≥ 14 mm, and peak E_2_ level: the correlation coefficients were 0.43 (*p* < 0.001) and 0.41 (*p* < 0.001), respectively.

### Cycle cancelation

**Figure 3:**
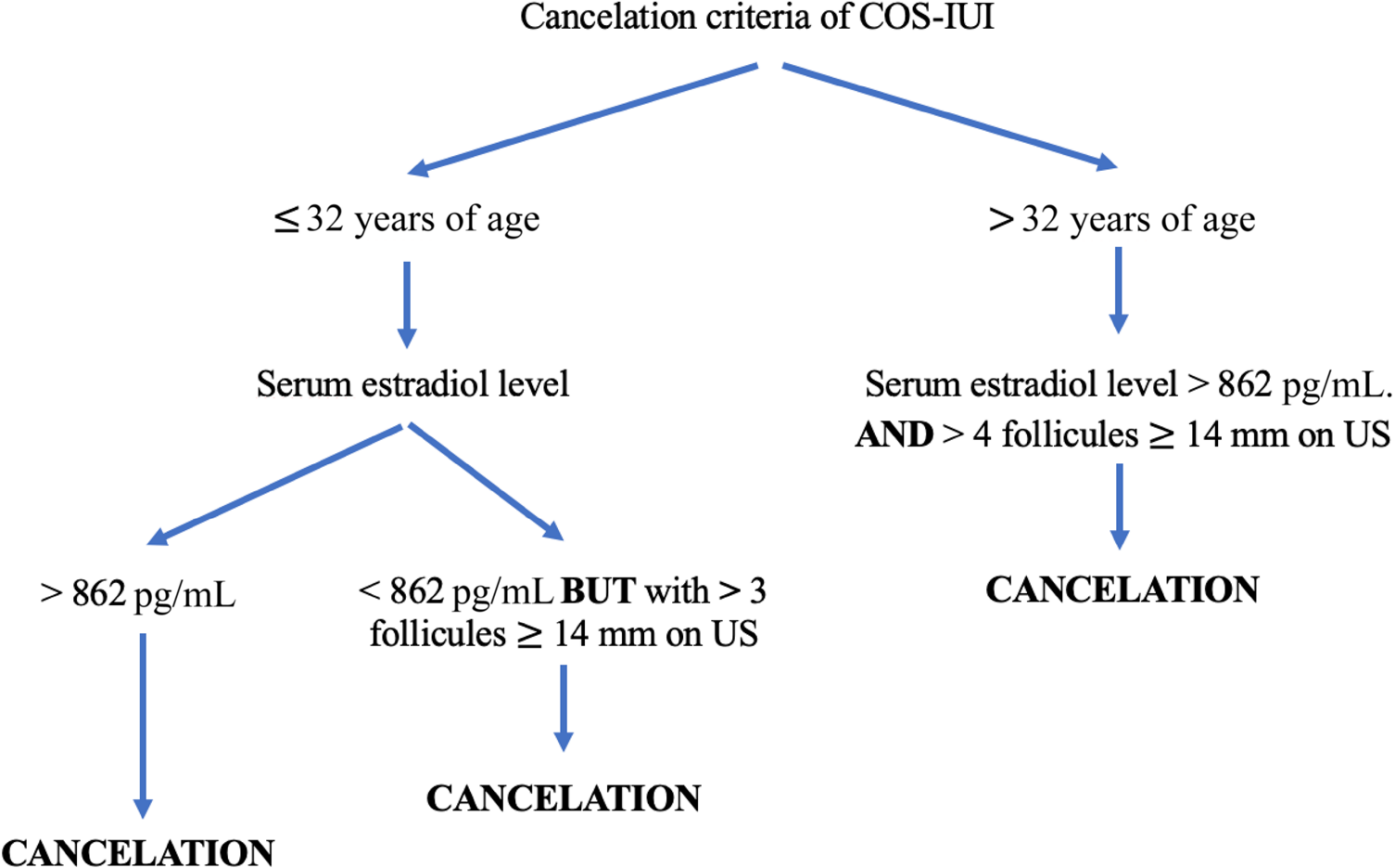
Algorithm for cycle cancelation in cases of excessive response to stimulation (inspired by Tur et al*.*).

We then analyzed the cycles canceled for excessive response to stimulation, and found that, in 57.5% (27/47) of cases, there was a concordance between a moderately elevated peak serum E_2_ level (> 600 pg/mL) and an excessive follicular response according to our algorithm (Fig. 3). Out of the 27 cases, 16 (59.3%) were canceled because there were 3 or 4 follicles ≥ 14 mm. In 9 cases of cancellation, there was a discordance between a normal follicular response and a high E_2_ level (> 900 pg/mL), while in 11 cases, there was a discordance between an excessive follicular response and lower than expected E_2_ level (< 600 pg/mL). Finally, 0.2% of cases (9/3630) were canceled for high peak E_2_ levels (> 900 pg/mL) associated to a non-excessive follicular response.

In 3 cases, the cycle cancelation criteria were not applied: (1) one patient < 32 years of age with one follicle between 10 and 14 mm and one follicle ≥ 14 mm, and an E_2_ level > 862 pg/mL. The patient ended up with a singleton pregnancy; (2) two patients < 32 years of age with serum E_2_ < 862 pg/mL but 4 follicles ≥ 14 mm, and both ended up with singleton pregnancies.

## Discussion

Our study has showed that the systematic measurement of peak serum E_2_ levels in COS-IUI cycles does not reduce the risk of multiple pregnancies when strict cancelation criteria based on the patient’s age and follicular monitoring are applied. We found that peak serum E_2_ was not predictive of the risk of MP with an area under ROC curve of 0.60 (0.52–0.69), and that the correlation with the number of follicles ≥ 10 mm and ≥ 14 mm was moderate (linear correlation r of 0.43 and 0.41, respectively.

In the past two decades, several studies have tried to identify the risk factors associated with twin and higher order multiple pregnancies (HOMP) in COS-IUI cycles, and develop prediction models that would allow to lower the MP rate without decreasing the overall success rates. Among these risk factors, serum E_2_ level has been found to be linked to MP rates, but only at very high levels and when combined with an excessive follicular response. In an analysis of 441 pregnancies following COS-IUI, out of which 9% were MP, Gleicher et al*.*^10^ found a significantly higher risk of MP when peak serum E_2_ level was > 1385 pg/mL or when there were > 6 pre-ovulatory follicles on US^10^. Tur et al*.*^12^ reported a 15.6% twin pregnancy and a 5.7% HOMP rate in 1878 pregnancies following COS-IUI. The HOMP rate was 19% when peak serum E_2_ was > 862 pg/ml with > 5 follicles > 10 mm in women ≤ 32 years of age^12^. The same authors reported in a later study that the use of a prediction model that includes the woman’s age, the number of preovulatory follicles, and the peak serum E_2_ level led to 285% reduction in the rate of HOMP^20^. By applying the same predictive model in our algorithm (Fig. 3), we found a twin pregnancy rate of 7.7% and a HOMP rate of 0.4%, both considerably lower than those cited. However, it should be noted that COS with 150 IU or higher of exogenous gonadotropins is considered a risk factor of MP when compared to COS with 50 or 75 IU^13,16^. The mean total FSH dose used by Tur et al*.* was 1120 IU compared to 744 IU in our study, and the mean number of follicles ≥ 10 mm was 4.7 compared to 2.3 in our study^12^. The use of lower gonadotropin doses and a strict predictive model allowed us to lower the MP rate while maintaining an acceptable overall clinical pregnancy (13.4%) and live birth rate (10.8%).

We aimed to assess whether the systematic measurement of peak serum E_2_ level, as per our algorithm, played a part in lowering the MP and HOMP rates. We found that serum E_2_ level was not a predictive factor of MP. Moreover, the Pearson coefficient showed only a moderate correlation between serum E_2_ level and the number of follicles ≥ 10 mm and ≥ 14 mm. On the other hand, multivariate analysis also found that the treatment duration, the gonadotropin doses used, and the number of follicles ≥ 10 mm and ≥ 14 mm were not predictive factors of MP. This could be the consequence of our adherence to strict cancelation criteria and the use of relatively low gonadotropins doses (50–100 IU) which limited the number of growing follicles. Indeed, there were only 3 cases (0.08%) where the cancelation criteria were not respected.

There were 9 cases (0.2%) that were canceled because the peak serum E_2_ level was very high (> 900 pg/mL), despite a normal ultrasound with no sign of excessive response to COS. Our rate is markedly lower than the 5.5% (68/1327) reported by Tur et al*.* in COS-IUI cycles^12^. Interestingly, the rate of HOMP in that study was 8% in women > 32 years of age and 12% in women ≤ 32 years of age^12^. In this instance, the measurement of peak serum E_2_ levels might have been useful, and prevented potential MP by signaling an excessive response to COS that was not suspected on US monitoring. However, the occurrence of these cases is low (0.2%).

**Figure 4:**
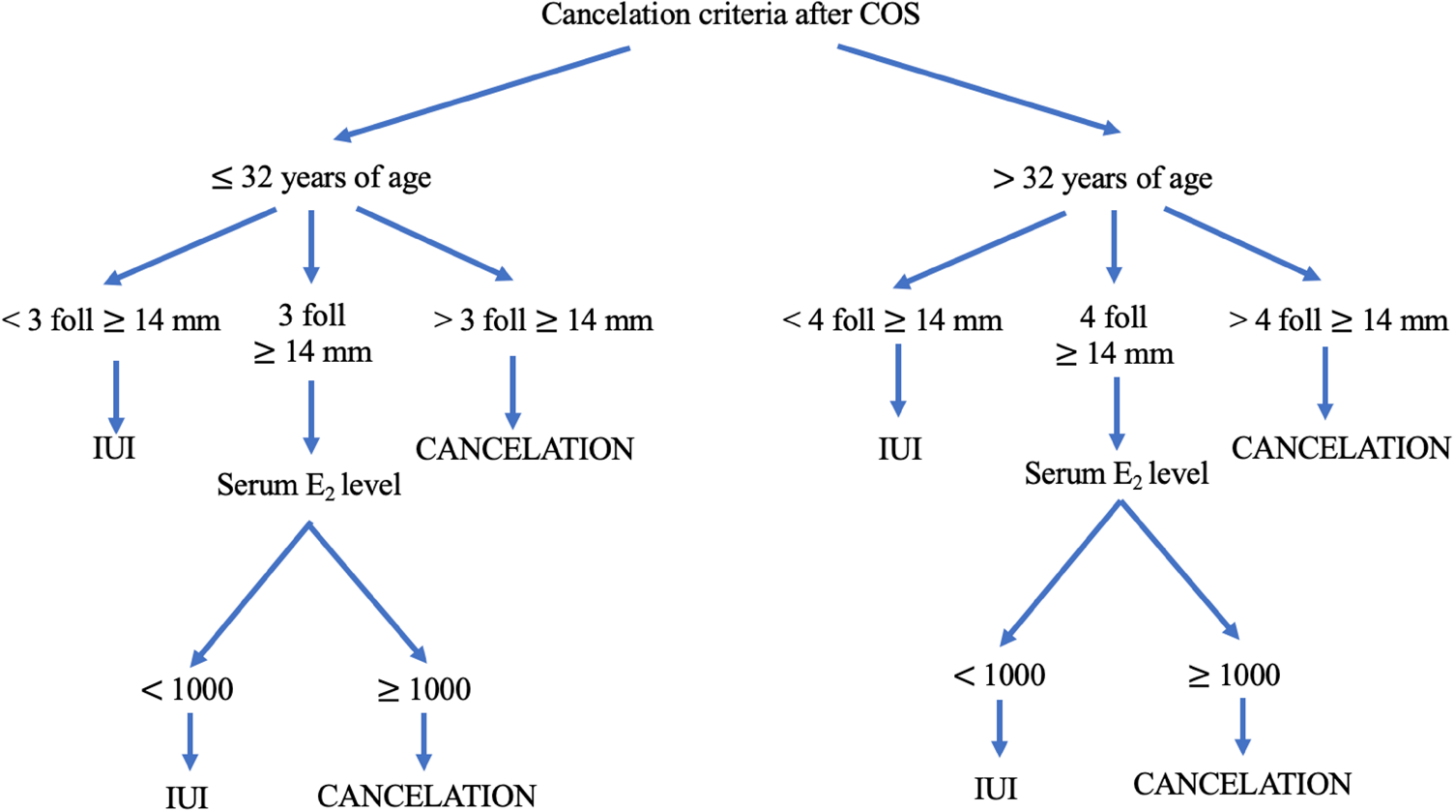
New algorithm for cycle cancelation of COS-IUI cycles.

Based on our findings, we decided to create a new algorithm for cycle cancelation that is more adapted to our current practice in COS-IUI cycles (Fig. 4). There are two major changes in the new algorithm: first, we modified the threshold for peak serum E_2_, and increased it from 862  to 1000 pg/mL. The initial threshold was based on the studies by Tur et al*.*^12,20^, who used radioimmunoassay (RIA) to measure serum E_2_ levels, while at our center, we use chemiluminescent immunoassay (CLIA). In general, the difference between these two methods is as follows: CLIA = 1.04 (RIA) + 20 pg/mL^21^. The calculated new threshold stands at 920 pg/mL, but we rounded it up to 1000 pg/mL to make it easier to use in daily practice. The second major modification is to abandon the systematic measurement of peak serum E_2_ levels, and only proceed with it in specific cases, depending on the US monitoring results. The new indications for serum E_2_ measurements are: 3 follicles ≥ 14 mm in women ≤ 32 years of age, and 4 follicles ≥ 14 mm in women > 32 years of age (Fig. 4).

The main limitation of our study is the retrospective design. It would be interesting to prospectively compare the two cancelation policies, one with and one without measurement of peak serum E_2_ levels. The main strength of our analysis in the inclusion of a large cohort of 3630 COS-IUI cycles over a period of 9 years. Moreover, and to the best of our knowledge, this is the first study assessing the role of serum E_2_ measurement in COS-IUI cycles when a strict cancelation policy is applied. The results of our analysis could be helpful for clinicians in their everyday practice.

In conclusion, our study has shown that, in COS-IUI cycles, when strict cancelation criteria based on the woman’s age and the number of growing follicles are used, the systematic measurement of peak serum E_2_ levels does not help reduce the rate of MP. Serum E_2_ levels could still help clinicians in the decision-making process in certain equivocal and problematic cases, when associated with the other criteria. In the near future, artificial intelligence models that incorporate all the risk factors of MP (woman’s age, number of growing follicles, type, duration and dose of ovarian stimulation, as well as serum E_2_ levels in certain cases) could calculate the risk of twin and HOMP in any given clinical situation, and help guide physicians and couples in deciding whether to proceed or cancel the treatment cycle.

## Methods

### Study setting

We performed a retrospective observational cohort study at the Angers University Hospital, a tertiary care center, between January 2011 and December 2019. The study was approved by the Ethics Committee of the Angers University Hospital (reference Number 2020/115). All methods were carried out in accordance with relevant guidelines and regulations.

### Participants

We included all patients who underwent Controlled Ovarian Stimulation with intrauterine insemination (COS-IUI) between 01/01/2011 and 21/12/2019 and who achieved a clinical pregnancy. Patients were pre-selected by the investigator and co-investigators from our database (Medifirst), which includes. All patients undergoing ART at our center, and contains all the relevant information, including the pregnancy outcomes. Informed consent was obtained from all subjects and/or their legal guardian(s).

The non-inclusion criteria were: patient’s refusal to be included in the study, ectopic pregnancies, first trimester miscarriage that occurred before the first ultrasound, the presence of a blighted ovum at the first ultrasound, and patients lost to follow-up following the positive pregnancy test.

### Procedures

All patients underwent the same COS-IUI protocol: injectable gonadotropins (recombinant or urinary) were started on day 3 of the menstrual cycle. The starting dose was based on the patient’s age, weight, ovarian reserve, and previous response to COS. According to these criteria, the starting doses used in our center were between 50 and 100 IU per day in 90% of cases. The first follow-up visit was scheduled 5 days later and included a pelvic ultrasound to measure the follicular diameter (the mean of the two largest diameters was retained) and endometrial thickness, as well as measurement of serum estradiol, LH, and progesterone levels. All ultrasounds were performed on a Voluson E8 machine (General Electric™, USA) using a high frequency (4–9 MHz) vaginal probe, and serum Estradiol was measured by immunoanalysis. (ADVIA Centaur^®^ XPT enhanced Estradiol (eE_2_) assay, Siemens™, Germany). When at least one follicle was ≥ 17 mm, ovulation was triggered with a sub-cutaneous injection of recombinant hCG (Ovitrelle^®^, 250 μg, Merck™, Lyon, France). IUI was performed 36 h after trigger. The patient’s partner provided the sperm sample at the center the morning of the insemination. The culture media used for sperm preparation was Ferticult™ (JCD laboratories, La Mulatière, France), and gradient centrifugation (PureSperm™, JCD Laboratoires, La Mulatière, France) was performed. IUI was performed in the outpatient clinic by one of our attending physicians or senior residents using a soft catheter (Elliocath^®^, Ellios Bio Tek, Paris, France). Micronized vaginal progesterone (Progestan^®^, Besins Healthcare, Paris, France), 200 mg twice daily, was used for luteal phase support. A serum Human Chorionic Gonadotropin (HCG) level was ordered 14 days after the insemination, and if positive, progesterone was continued until 7 weeks gestational age when an ultrasound was performed to confirm the pregnancy.

Cycles with an excessive response to stimulation were canceled. The cancelation criteria, based on the studies of Tur et al.^12,20^ are detailed in Fig. 3. Serum E_2_ levels on trigger day were included in the cancelation criteria, along with the patient’s age and the number of follicle ≥ 14 mm.

### Outcomes

Our main outcome measure was the area under Receiver-Operating Characteristic (ROC) curve for serum E_2_. Our secondary outcome measures were the clinical pregnancy (CP) (defined as a positive fetal heartbeat at 7 weeks GA) rate, the multiple pregnancy (MP) (defined as the presence of more than one embryo on the ultrasound) rate, the miscarriage (defined as the loss of a confirmed intrauterine pregnancy before 20 weeks GA^21^ rate, and the live birth (LB) (defined as the birth of a viable baby > 25 weeks GA) rate.

In order to assess the potential predictive factors of MP, we also analyzed the following criteria: patient’s age, the stimulation protocol (total dose and days of stimulation), the cause of infertility, the number of motile sperm inseminated, and the number of per-ovulatory follicles ≥ 10 and ≥ 14 mm.

All data were recorded from an electronic case report form (eCRF) specifically elaborated for the study (eCRF CleanWEB, Telemedicine Technologies S.A.S), and were collected and stored in a REDCap database (REDCap 8.5.19 Vanderbilt University, Nashville, USA) hosted by the Clinical Research Center of the Angers University Hospital.

### Statistical analysis

Qualitative variables were expressed as numbers and percentages, and compared using the Pearson chi-squared or Fisher's exact test. Quantitative variables were expressed as means and standard deviations, or median and percentiles (25 and 75), and compared using Student’s t test or Mann–Whitney’s non parametric test. The predictive value of serum E_2_ levels for the MP rate was analyzed using the ROC curve. On the other hand, and in order to take into account the nonindependence of observations (since a patient can be included multiple times), a univariate Generalized Estimating Equation (GEE) model was used to compare the singleton and MP rates and a multivariate GEE model including the mean E2 and factors with a p-value less than 0.20 in univariate analysis (total treatment dose, duration of treatment, and number of follicles ≥ 10 mm or ≥ 14 mm) was used to take into account the effect of potential confounding factors. Finally, the correlation between serum E_2_ levels and the number of pre-ovulatory follicles was assessed using Pearson’s r correlation coefficient. All analyses were performed using SPSS version 22.0 (New York, USA). A *p*-value < 0.05 was considered statistically significant.

## Supplementary Information

Supplementary Information.

## Data Availability

The dataset generated during the current study can be made available upon request to the corresponding author.
