# Supplementary material for: The role of peak serum estradiol level in the prevention of multiple pregnancies in gonadotropin stimulated intrauterine insemination cycles
Source: Sci Rep. 2022 Nov 15;12:19554. doi: 10.1038/s41598-022-23470-9 (PMC9666543; doi:10.1038/s41598-022-23470-9)
Supplement: Supplementary file 1 — Supplementary Information. [file 41598_2022_23470_MOESM1_ESM.docx]

**Supplementary data.** Multivariate logistic regression for MP (twin + Higher Order Multiple Pregnancies) (Multivariate GEE model).

|  | aOR (95% CI) | p-value |
| --- | --- | --- |
| Duration | 1.04 (0.96-1.27) | 0.31 |
| Doses | 0.99 (0.94-1.06) | 0.99 |
| Serum Estradiol levels | 1.07 (0.86-1.35) | 0.52 |
| Follicles ≥ 10 mm on trigger day | 1.17 (0.87-1.58) | 0.24 |
